# Juvenile idiopathic arthritis with systemic onset with inflammatory bone lesions: two case reports of patients successfully treated with canakinumab and experience gained from literature

**DOI:** 10.3389/fped.2023.1163483

**Published:** 2023-05-31

**Authors:** Ekaterina I. Alexeeva, Tatyana M. Dvoryakovskaya, Irina T. Tsulukiya, Natalia M. Kondrateva, Natalia M. Solomatina, Gleb V. Kondratiev, Luliia V. Peshekhonova, Mikhail M. Kostik

**Affiliations:** ^1^ Department of Rheumatology, National Medical Research Center of Children's Health, Moscow, Russia; ^2^ Russian Federation Department of Pediatrics, Sechenov First Moscow State Medical University, Moscow, Russia; ^3^ Association of Pediatric Rheumatologists, Moscow, Russia; ^4^ Pediatric Pulmonology, Saint-Petersburg State Pediatric Medical University, Saint-Petersburg, Russia; ^5^ Pediatric Oncology, Saint-Petersburg State Pediatric Medical University, Saint-Petersburg, Russia; ^6^ Hospital Pediatry, Saint-Petersburg State Pediatric Medical University, Saint-Petersburg, Russia

**Keywords:** chronic non-bacterial osteomyelitis 1, juvenile idiopathic arthritis with systemic onset 2, canakinumab 3, autoinflammation 4, chronic recurrent multifocal osteomyelitis 5, interleukine-1 6

## Abstract

Non-bacterial osteomyelitis (NBO) is a rare chronic inflammatory bone disease related to immune system dysregulation. This disease belongs to a family of autoinflammatory diseases. It often coexists with other TNF-α-mediated immune-mediated diseases such as juvenile idiopathic arthritis (JIA) and inflammatory bowel diseases. Previously, interleukin-1-driven inflammation was described predominantly in monogenic cases of NBO, such as DIRA syndrome or Majeed syndrome. However, the association between NBO and JIA with systemic onset (soJIA) has not been described yet.

Herein, we describe the cases of two patients with soJIA with inflammatory bone lesions wherein canakinumab (anti-interleukin-1β antibodies) caused remission.

**Case descriptions:**

Patient 1–A 6-month-old boy with typical soJIA suffered a destruction of the 7th to 9th ribs and the left pubic bone. Antibiotics, IVIG, and cyclosporine proved ineffective. Corticosteroids were effective, but due to the factor of corticosteroid dependence, which has some disadvantages, canakinumab with a dosage of 4 mg/kg was initiated every 4 weeks, which completely controlled the disease and allowed to taper corticosteroids.

Patient 2—A 2-year-old girl developed chronic non-bacterial osteomyelitis of the 5th rib 2 months after taking corticosteroids prescribed for typical soJIA. She underwent surgical debridement removal, and several courses of antibiotics proved ineffective. She developed macrophage activation syndrome, following which anakinra was prescribed, which resulted in only temporary improvement. Therefore, this drug was switched to canakinumab, which caused corticosteroid-free remission.

**Conclusion:**

This is the first description of a rare association of soJIA with inflammatory bone lesions with the proven efficacy of IL-1 blockade. The association of two autoinflammatory conditions should indicate IL-1-driven mechanisms and a possible genetic basis. Follow-up genetic and functional studies are required to better understand the pathogenesis of such overlapping diseases.

## Introduction

Non-bacterial osteomyelitis (NBO) is a rare chronic inflammatory bone destructive disease with unknown etiology (1). NBO belongs to a family of autoinflammatory diseases (2). Innate immune system dysregulation is the basis of its pathogenesis (3, 4). Several previous studies demonstrated the hyperproduction of IL-1β, TNF-α, IL-18, and IL-17a, with a lack of production of anti-inflammatory cytokines, such as IL-10, IL19, and IL-4 [(5–8]). NBO is often associated with TNF-α-mediated diseases such as juvenile spondyloarthritis, psoriatic arthritis, and inflammatory bowel diseases (9). According to the ARC/CARRA treatment plan, TNF-α inhibitors should be used in cases where non-biologic DMSRDs fail (10). There are several monogenic forms of NBO such as DIRA syndrome and Majeed syndrome, with a hyperproduction of IL-1 and successful treatment with IL-1 inhibitors, but the number of such cases is few (11, 12). In real clinical practice, IL-1 inhibitors are used rarely compared with TNF-α inhibitors (9).

Juvenile idiopathic arthritis with systemic onset (soJIA) is a typical autoinflammatory disease with a hyperproduction of several cytokines such as IL-1, IL-6, IL-18, TNF-α, IL-17A, interferon *γ*, and others (13, 14). Despite the broad spectrum of cytokines operating in soJIA pathogenesis, only the inhibition of IL-1 and IL-6 has shown the best results (15–17). The inhibition of TNF-a has partial efficacy and works better in patients in whom systemic features have disappeared and the disease has taken a chronic articular course (18).

Compared with NBO, soJIA is a rare autoinflammatory disease associated with other rheumatic diseases. Sometimes in the literature, the soJIA phenotype was described in autoinflammatory diseases (AID), e.g., TRAPS syndrome or CAPS syndrome (19, 20). However, the association between soJIA and NBO has not been described yet.

Herein, we describe the cases of two patients with soJIA overlapping with NBO and who were successfully treated with the IL-1β inhibitor, canakinumab.

## Case description

**Figure 1:**
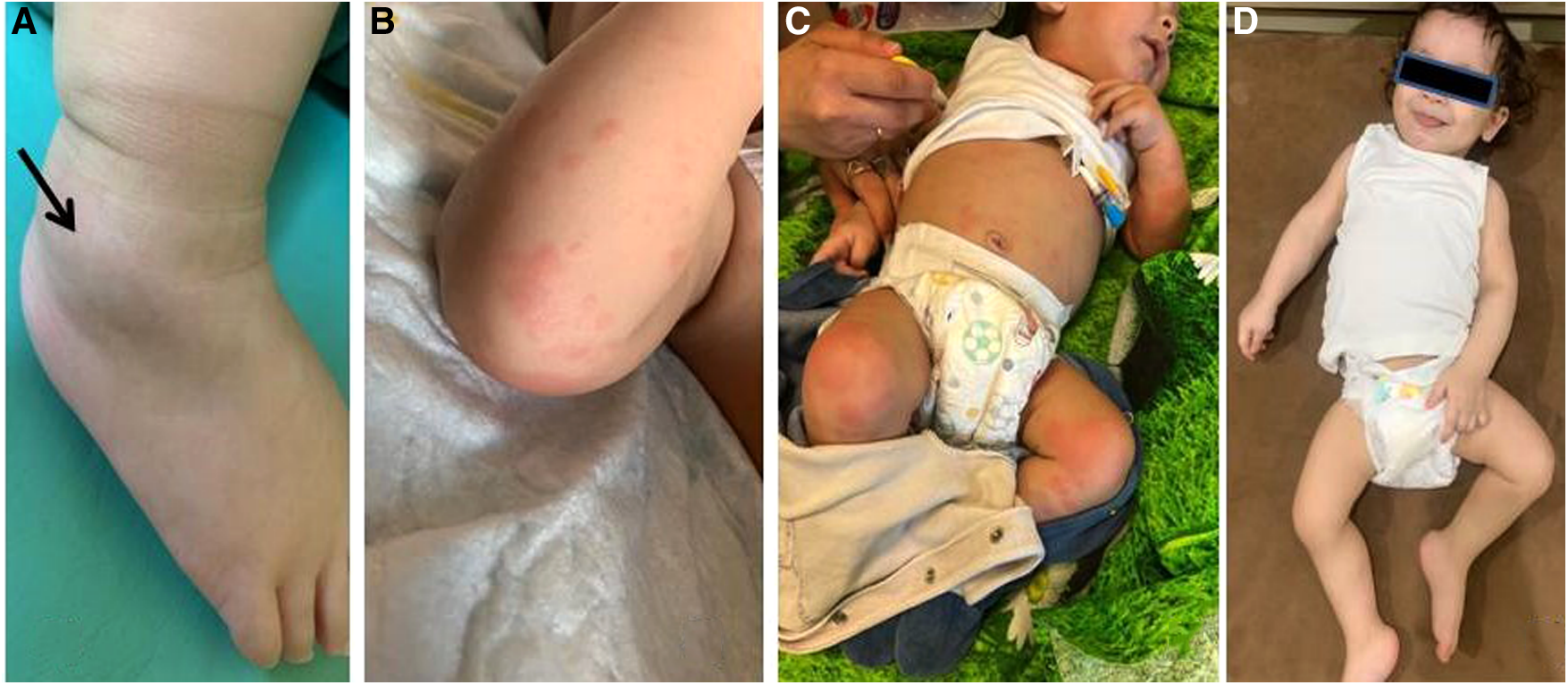
Patient 1: (**A**) right ankle swelling (arrow); (**B**) typical maculopapular rash; (**C**) the patient’s appearance before (rash and severe general condition of the patient) and (**D**) 3 months after (no rash, improvement of general condition) canakinumab treatment.

**Patient 1** was a boy who contracted the disease at 6 months of age with spiking fever, ankle arthritis, and a typical maculopapular rash associated with fever spikes (Figures 1A–C). He had anemia (Hb 77 g/L; n.v. 110–135 g/L), neutrophilic leukocytosis (WBC 28 × 109/L; n.v. 6–15 × 109/L), thrombocytosis—773 × 109/L (n.v.150–480 × 109/L)), ESR 74 mm/h (2–20 mm/h), increased CRP—136 mg/L (n.v. < 5 mg/L), increased ALT—92 U/L (n.v. < 40 U/L), AST—120 U/L (n.v. < 40 U/L), LDH—441 U/L (n.v. 91–295 U/L), ferritin—1,306 ng/ml (n.v. 12–327 U/L), and triglycerides 5 mmol/L (n.v. 0.34–1.6 mmol/L).

An examination revealed lymphadenopathy (supraclavicular, axillar, mesenteric, and retroperitoneal), arthritis of both hips and soft tissue swelling surrounding both ankles. Viral and bacterial infections and malignancy were ruled out, and empiric antibiotic therapy proved ineffective. This led to a diagnosis of soJIA with MAS, and the patient was started on systemic corticosteroids, cyclosporine A, and IVIG. All features of the disease disappeared and laboratory tests showed normal results. The patient was discharged from the hospital with a recommendation of corticosteroid tapering and continued cyclosporine A.

One month later, the patient developed a flare: spiking fever, typical rash, and knee, elbow, and hip arthritis with similar laboratory findings as at onset.

**Figure 2:**
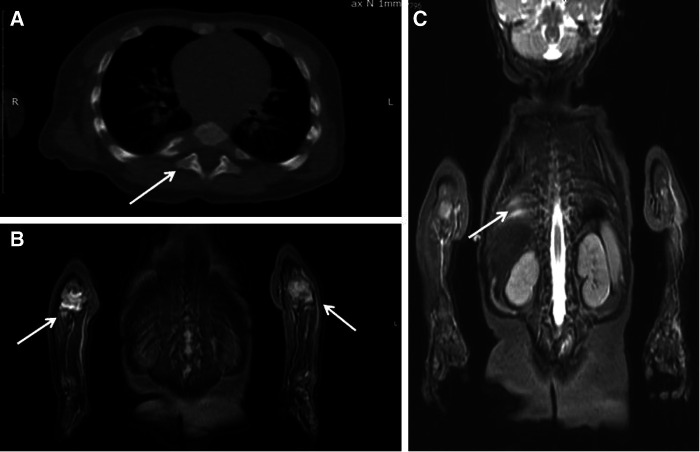
Patient 1: (**A**) chest CT: the destruction of the head of the 7th right rib (arrow). (**B,C**) Whole-body MRI (COR STIR sequence) Bone marrow edema of the 8th and 9th right ribs (**C**), synovitis of both elbows (**B**).

A chest CT taken after a prolonged fever revealed 7th right rib destruction with a swelling of the surrounding tissues and a focal destruction of the left pubic bone close to symphysis (Figure 2A). These findings were absent in CT and MRI done 1 month previously. Several courses of IV antibiotics and antimycotics proved ineffective. Pubic bone biopsy revealed signs of chronic osteomyelitis (infiltration with lymphocytes, plasma cells and monocytes, and bone sclerosis), which was confirmed by a second opinion sought from an alternative center. Further investigations in the following year (whole-body MRI) revealed bone marrow edema of the 8th and 9th right ribs and the left pubic bone, soft tissue swelling on the medial part of the left hip, and arthritis of the 7th costovertebral joint, elbows, hips, shoulders (Figures 2B,C).

Whole-exome sequencing was done, which revealed a heterozygous c.2282G > A (R761H) MEFV variant. This variant is characterized as “likely pathogenic”. The results of basic immunology tests (immunoglobulin levels, T-/B-/NK-cell counts, and oxidative burst test) were unremarkable.

The presence of sJIA with MAS, MEFV variant, and failure of previous non-biologic DMARD led to the initiation of canakinumab at a dosage of 4 mg/kg every 4 weeks. When the canakinumab course was under way, the features of the disease in the patient disappeared (Figure 1D), which was proved by normal laboratory test results, following which the dosage of corticosteroids was reduced to 0.125 mg/kg.

**Figure 3:**
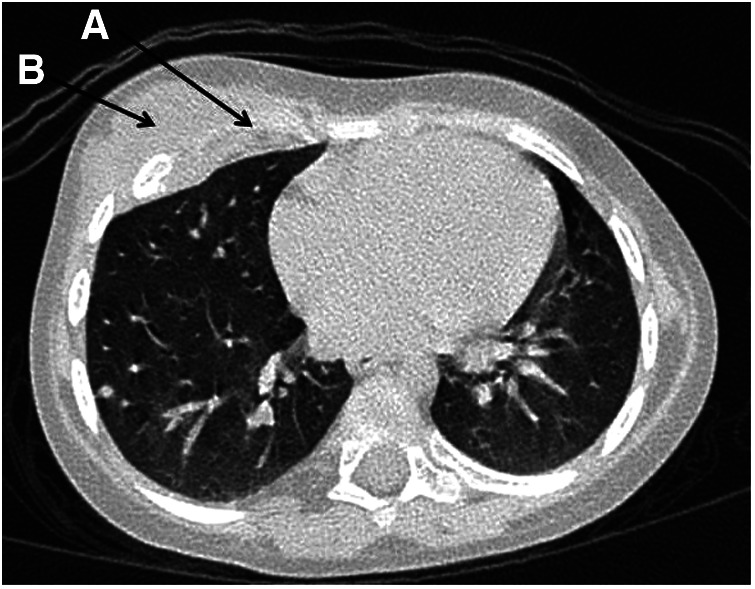
Patient 2: the destruction of the 5th right rib (**A**) and a mass lesion of the right anterior chest wall (**B**).

**Patient 2.** A 2-year-old girl developed high spiking fever, rash (flat, non-itchy salmon-pink macules and papules coinciding with fever), arthritis, leukocytosis with neutrophilia, thrombocytosis, increased CRP, ESR, anemia, hyperferritinemia, and increased LDH with a positive effect of systemic corticosteroids. Two months later when the corticosteroids were tapered, she developed a mass lesion of the right anterior chest wall with axillar lymphadenopathy. A repeated chest CT scan revealed a destruction of the 5th right rib with periosteal reaction and an extraosseous mass lesion 25 × 48 × 56 mm under the musculus pectoralis major with foci of contrast uptake (Figure 3). Two surgeries were performed. Specific infections (tuberculosis and mycosis) and malignancy were ruled out. A morphological examination revealed chronic bone inflammation (infiltration with lymphocytes, plasma cells with a small count of neutrophils, and bone sclerosis). A CT revealed rib osteosclerosis. The fever and rash persisted, indicating the poor efficacy of antibiotics. Episodical corticosteroid injections for rash reduced the fever and rash. After 5 months the girl was admitted to our department of immunology and allergology, where she again developed fever and rash, leukocytosis, neutrophilia, and thrombocytosis-increased CRP. Due to possible immunodeficiency, antibiotics were started again, but her condition worsened. She developed a highly persistent non-spiking fever and rash and also a full-blown macrophage activation syndrome—WBC 4.6 × 109/L; platelets 135 × 109/L; Hb 66 g/L, CRP 59 mg/L, AST 105 U/L, LDH 1,263 U/L, ferritin 260 ng/ml, D-dimer 2,486 ng/ml (n.v. < 500), fibrinogen 1.4 g/L). The known immune deficiency was ruled out, and WES did not reveal any relevant findings. Antibiotics were discontinued, and whole-body MRI, chest, and abdominal CT revealed axillar, abdominal, mesenteric, inguinal lymphadenopathy, hepatosplenomegaly, joint effusion, and 5th rib osteosclerosis. There were no lung infiltrates. Due to the inefficacy of antibiotics and the positive effect of corticosteroids administered earlier, anakinra with a dosage of 5 mg/kg/day was initiated, which had good efficacy. In a span of 2 weeks, the patient developed a flare, and in the following 2 weeks, anakinra was switched to canakinumab at a dosage of 4 mg/kg every 4 weeks, which resulted in a complete resolution of the disease. Now she is in remission upon canakinumab treatment.

## Discussion

Herein, we describe two cases of patients with soJIA with inflammatory bone lesions. To our knowledge, this is the first description of such a combination of two conditions with autoinflammatory mechanisms. Both cases show similarities in terms of young onset age, initial involvement of the plane bones, initial features of sJIA, and efficacy of the IL-1β inhibitor canakinumab. Typical NBO starts in preschool-age children with low systemic inflammation (low-grade fever, low CRP, no leucocytosis, and no thrombocytosis) (2). An extremely young onset age (before 1 year) is usually a hallmark of the monogenic origin of the disease (e.g., DIRA syndrome) or primary immunodeficiency syndrome (chronic granulomatosis disease). The pattern of bone involvement in our patients bore a close resemblance with bone involvement in monogenic autoinflammatory diseases, such as DIRA-syndrome or Majeed syndrome, because of very young onset age and association with systemic inflammation. We feel that “soJIA with inflammatory bone lesions” is the more appropriate term for the diagnosis of our patients, because their bone inflammation does not correspond to classical NBO. Both patients are fit candidates for performing whole-genome sequencing to find the possible candidate genes, due to their young onset age and features, which are similar to those of monogenic autoinflammatory diseases.

The efficacy of IL-1 inhibition serves as an indirect confirmation of clear autoinflammatory mechanisms in the pathogenesis of the diseases (21). The known monogenic forms of NBO - the DIRA syndrome or osteomyelitis, sterile multifocal, with periostitis and pustulosis; OMPP (OMIM: 612852) is a rare inherrited disorder caused by homozygous mutation in the IL!RN (Interleukin 1 receptor antagonist) gene (147679). The loss of IL1RN function results in the activation of Interleukin 1 receptor signaling, and its phenotype resembles chronic non-bacterial osteomyelitis (11). The inhibition of IL-1β signaling with canakinumab limits the progression of sterile osteomyelitis.

Classical NBO usually required TNF-α inhibitors, and the outcomes of anti-IL-1 treatment are limited. After a PubMed search, 27 manuscripts on interleukin-1 antagonists were found, predominantly anakinra (*n* = 19) and rare canakinumab (*n* = 7), and one manuscript was discovered on rilonacept in six DIRA patients (22).

The closest to soJIA is adult-onset Still’s disease. It has a similar IL-1 upregulation pathogenesis, which was confirmed by an identical transcriptional profile (23). There is only one published case on the overlapping of adult-onset Still’s disease with chronic recurrent multifocal osteomyelitis with a complete remission of anakinra (24).

The outcomes of anti-IL-1 treatment are predominantly limited to monogenic autoinflammatory diseases such as DIRA or Majeed syndrome (25).

In the biggest case series of anakinra in chronic non-bacterial osteomyelitis, unresponsiveness to NSAIDs and bisphosphonate improvement were achieved (decreasing VAS, ESR, CRP, and number of lesions), but new lesions occurred and some of them were symptomatic (26) In this study, there were no patients who were unresponsive to TNF-α inhibitors before anakinra administration. The data on canakinumab are scarce and limited to seven manuscripts on 10 patients. The cumulative data are presented in Table 1.

**Table 1:** A review of interleukin-1 blockade treatment for non-bacterial osteomyelitis.

Reference	Case description	Age of		Outcomes
		onset	diagnosis	
(27)	Two brothers (A and B) with Majeed syndrome failed to benefit from corticosteroids and etanercept, and they had recurrent fever, thrombocytosis, dyserythropoietic anemia, high CRP, and CRMO course.			CR after anakinra and CNKB 4 mg/kg Q4W in both siblings.
	A) tibiae, left fibula, radii, and left lower ribs.	3 mo	13 mo	
	B) both tibiae, left fibula, and left radius and ulna; multiple phalangeal bones, right humerus, elbows, knees, and ankles. Synovitis of the right ankle and left knee and temporary flexion contracture of the right knee.	6 mo	29 mo	
(12)	1) Girl with Majeed syndrome. Involvement of left ankle arthritis, bone ache, arthralgia, CRMO course, dyserythropoietic anemia, high CRP—ESR	4 y	14 y	CR on CNKB 4 mg/kg Q4W
	2) Boy with CNO. He had arthritis, back pain, tender right clavicle, arthralgia, CRMO course, anemia, and high CRP-ESR. He failed to benefit from previous treatment with prednisone, pamidronate, infliximab, and methotrexate	7 y	7 y	CNKB Q8W with initial CR followed Q6W due to a lack of efficacy (partial remission)
	3) Boy with CNO. Bone ache, arthralgia, and CRMO course. He failed to benefit from naproxen, pamidronate, CNKB, and methotrexate. He had anemia and high CRP-ESR.	12 y	13 y	Initial CR of CNKB with loss of efficacy and switched to infliximab
(28)	Girl with mevalonate kinase deficiency with fever, rash, lymphadenopathy, hepatosplenomegaly, anemia, increased inflammation, recurrent infections, and a single episode of hip involvement, evaluated as osteomyelitis.	14 mo	14 mo	Anakinra, followed by CNKB; both encourage CR.
(29)	A 12-year-old girl with DIRA syndrome with systemic inflammation, respiratory distress, joint swelling, pustular rash, multifocal osteomyelitis, and periostitis. Failed to benefit from prednisolone, etanercept, and sulfasalazine.	12 y	24 y	Failed to benefit from CNKB. CR with adalimumab and colchicine.
(30)	Boy with DIRA with fever, serositis, pancreatitis, and high inflammatory markers; multifocal pelvic bone inflammation suggestive of CRMO.	13 mo	3 y	CR on anakinra, switched to CNKB with flare in 6 weeks. Restart of anakinra with CR.
(31)	Boy with DIRA syndrome, presented with recurrent episodes of systemic inflammation with severe disabling osteomyelitis (pelvic, hip, ribs) with mild pustular skin rash.	4 mo	7 y	CNKB 2 mg/kg Q4W with initial efficacy, flared after 7th dose, switched to anakinra with CR
(32)	A 13-year-old girl with a multidrug-resistant and pyoderma gangrenosum–complicated CRMO.		** **	Initial CR on CNKB, followed by decreased efficacy.

CNO, chronic non-bacterial osteomyelitis; CNKB, canakinumab; CR, complete remission; CRMO, chronic recurrent multifocal osteomyelitis; mo, months; Q4W, every 4 weeks, Q6W, every 6 weeks; Q8W, every 8 weeks, y, years.

Canakinumab showed complete remission in 5/10 (50%) of patients, partial remission in 1/10 (10%), and ineffective remission in 4/10 (40%), with initial short-term efficacy in three children.

## Conclusion

This study is the first description of a rare association of soJIA with inflammatory bone lesions with the proven efficacy of IL-1 blockade. The association of two autoinflammatory conditions should indicate IL-1-driven mechanisms and a possible genetic basis. Follow-up genetic and functional studies are required to better understand the pathogenesis of such overlapping diseases.

## Data Availability

The original contributions presented in the study are included in the article; further inquiries can be directed to the corresponding author.
